# Analysis of a nurse-provided on-call peritoneal dialysis support in an outpatient reference care centre

**DOI:** 10.1186/s12912-024-01812-4

**Published:** 2024-03-01

**Authors:** Annemarie Albert, Stefan Richter, Philipp Kalk, Philipp Stieger, Rainer Peter Woitas, Rüdiger C. Braun-Dullaeus, Christian Albert

**Affiliations:** 1 Diaverum Renal Services, Am Neuen Garten 11, Potsdam, 14469, Germany; 2 Department of Nephrology and Endocrinology, Ernst von Bergmann Hospital, Charlottenstraße 72, Potsdam, 14467 Germany; 3 University Clinic for Cardiology and Angiology, Otto-von-Guericke University Magdeburg, Leipziger Str. 44, Magdeburg, 39120 Germany; 4 Department of Nephrology, Central Clinic Bad Berka, Robert-Koch-Allee 9, Bad Berka, 99438 Germany

**Keywords:** Peritoneal dialysis, Home therapy, On-call support, Renal replacement therapy, Telemedicine, Adverse event, Nursing

## Abstract

**Background:**

To analyse the nature of medical or technical emergency issues of ambulatory peritoneal dialysis (PD) patients calling a nurse-provided emergency PD support service of a reference centre that is provided all year in the after-hours.

**Methods:**

We retrospectively analysed patients’ chief complaint, urgency, resolution of and association to current PD treatment and modality directed to an on-call nurse-provided PD support service from 2015–2021 based on routinely collected health data. Calls were systematically categorized being technical/procedural-, medical-, material-related or type of correspondence. Call urgency was categorized to have “immediate consequence”, inquiry was eligible for “processing next working day” or whether there was “no need for further action”. Call outcomes were classified according to whether patients were able to initiate, resume or finalize their treatments or whether additional interventions were required. Unexpected adverse events such as patients’ acute hospitalization or need for nurses’ home visits were evaluated and quantified.

**Results:**

In total 753 calls were documented. Most calls were made around 7:30 a.m. (5:00–9:00; median, 25-75^th^ CI) and 6:30 p.m. (5:00–8:15). 645 calls were assigned to continuous ambulatory- (CAPD) or automated PD (APD). Of those, 430 calls (66.7%) had an “immediate consequence”. Of those 77% (*N* = 331) were technical/procedural-, 12.8% (*N* = 55) medical- and 6.3% (*N* = 27) material related issues. 4% (*N* = 17) were categorized as other correspondence.

Issues disrupting the course of PD were identified in 413 cases. In 77.5% (*N* = 320) patients were able to initiate, resume or finalize their treatment after phone consultation. Last-bag exchange was used in 6.1% enabling continued therapy in 83.6%.

In 35 cases a nurse visit at patients’ home or patients' visit to the practice at the earliest possible date were required, while hospitalization was required in seven medical category cases (5.4% and 1.09% of total assessed calls, respectively).

**Conclusion:**

The on-call PD-nurse provides patient support for acute and imminent issues enabling them to successfully initiate, resume or finalize their prescribed treatment. Nurses triage of acute conditions facilitated rapid diagnostics and therapy. Maintaining quality PD homecare, the provision of trained personnel is indispensable. The information gathered in this study may therefore be used as a foundation to tailor educational programs for nephrology nurses and doctors to further develop their competencies in PD.

**Supplementary Information:**

The online version contains supplementary material available at 10.1186/s12912-024-01812-4.

## Background

Peritoneal dialysis (PD) is a widely used renal replacement therapy allowing end stage renal disease patients to undergo a home-based treatment [1, 2].

The development and maintenance of appropriate patient therapy-support is mandatory to provide safe and effective home-based care [3]. Maintaining therapy-support requires a well-trained PD-team of qualified nurses and physicians, skilled in the principles of clinical, technical, and procedural practice of continuous ambulatory- (CAPD) or automated PD (APD) modalities and management of potential complications [4]. Providing all year 24-h therapy-support is recommended, as a majority of patients perform PD outside of standard office hours [5, 6]. The important role of telephone triage in the initial contact with the patient is traditionally provided by nurses. Such customized, continuous, prompt and decisive on-call support is essential to the provision of optimal home-based care, eventually improving patients' acceptance of PD-modality, satisfaction, overall feeling of safety and outcome in case of emerging issues [7, 8].

However, a recent study from Germany showed, that many outpatient renal centres care for a few PD patients, only [9, 10]. As a result, it may be difficult for many nurses to acquire practice routine in this area of expertise. In the event of incident, uncertainties and fears among many on-call nurses are observed [11]. Consequently, PD support may eventually get outsourced to private- or vendor-provided services with potentially less favourable outcome [12].

The nursing staff of a PD-centre may yet be most familiar with their patients’ individual insecurities and underlying health conditions [7]. To counteract insecurities and improve the knowledge and quality of care, the establishment of training programs should be considered to prepare nurses for the nature of emerging incidents which may come across in on-call duty shifts [13].

However, limited information is available on home-dialysis incidents [14–16] and no previous study characterized the subject and management of complications in a nurse-provided on-call PD support in the after-work hours. Such information is important to enable or improve the development of training programs for PD nurses empowering them to confidently perform in on-call duty assignments. Accordingly, the aim of this study was to systematically determine and analyze the nature of emergency on-duty PD service calls in our outpatient PD reference centre.

## Methods

### Foundation of patient training in our centre

In our centre, experienced PD nurses provide individualized patient care, training and education of the performance of APD and CAPD in the patients’ homes, while following an underlying set of standards. This will include the training for hygiene measures and, typical pitfalls of the procedures. Prevention and thereby reducing infections due to touch contamination is the hallmark of a successful PD program [17]. Such training usually takes 3–5 days. If patients are experiencing repeated issues, we invite them for a refresher course.

### On-call PD support service

Generally, patients will direct their elective concerns to the PD staff during office hours using the PD bureaus’ number. On-call PD support service in our centre is provided all year and realized using a cell phone which the on-duty PD nurse carries on their person any time from 4:00 p.m. to 07:00 a.m. on weekdays, and 24 h during the weekend or official holidays. Staff is typically assigned in 1-week blocks.

The service cell phone number is given to all patients when beginning the PD home-training. It is also provided by a recorded message on a telephone responder when calling the PD bureau. Additionally, when staying on the line, callers will automatically be forwarded to the on-duty support in the after-hours, on weekends or official holidays. Patients do not have direct phone access to the on-call nephrologist; thus, all issues are initially triaged by the on-call nurse who may then consult the nephrologist or direct the call if needed.

### Data collection and categorization

Details of all calls were prospectively documented in short, standardised protocols including complete years 2015–2021 by the nurse on duty for their respective shifts. For this retrospective analysis of routinely collected clinical patient record data three readers, two nephrologists (AA and CA) and the head nurse (SR) independently reviewed all call protocols, extracted and systematically categorized documentation according to time and date, issue or chief complaint, resolution of and association to current PD treatment or modality. For all calls we determined the grade of urgency and if they required any further action or assistance to successfully initiate, resume or finalize a treatment session. Discrepancies were resolved by consensus.

According to protocol, the extracted data was anonymized immediately upon access to the patients’ clinical record and no other than retrospective routine clinical data was collected or analysed.

Calls were categorized being 1) technical issues concerning APD machine setup or being alarm-related, which included any equipment malfunction, APD machine alarms or error codes preventing to initiate, resume or finalize a treatment session; prescription-, software- or periphery related settings and errors, such as confirmation of prescription updates or external modem connectivity failure. Procedural assistance for CAPD was also assigned to this category.

2) major medical issues with a potential for harm, which included (but was not limited to) suspected peritonitis (abdominal pain, cloudy drainage, fever, diarrhoea, vomiting), bloody drainage, suspected catheter dislocation or exit site leakage, severe or refractory hypertension or hypotension, weight fluctuations with oedemas or breathing discomfort.

3) material-related such as insufficient or defective bags, CAPD- and APD-supplies or dressing material or handling errors leading to unsterile PD-catheter transfer set.

4) correspondence being general clinical/nursing related, which included (but was not limited to) general medical questions regarding medication, practice visits or psychosocial support as may be needed for patient reassurance (counselling) and “medical correspondence” with hospital- or outpatient practice-staff.

A sequential number was assigned to each call and patient, respectively. Calls from relatives or hospital staff who provided relevant health information on behalf of the patient or performed assisted PD were assigned to the respective patient ID-number.

As many patients switched PD-modalities during the observation timeframe we limited the present analysis to a per-call assessment and refrained from additional per-patient analysis.

### Materials used for peritoneal dialysis

For APD homechoice Claria with sharesource remote connectivity (Baxter, BX, Unterschleißheim, Germany) and sleep-safe harmony cyclers with PatientOnLine software (Fresenius Medical Care, FMC, Bad Homburg, Germany) were used as FMC Home Bridge remote connectivity is not yet available in Germany. Physioneal, nutrineal, icodextrin solutions (BX) or stay safe- and sleep safe balance solutions (FMC) were prescribed by the treating nephrologists independently of this investigation.

### Outcome measures

Call urgency was categorized to have “immediate consequence”, inquiry was eligible for “processing next working day” or whether there was “no need for further action” including non-urgent calls with non-treatment-related issues that could easily have been deferred to regular office-hours. For all calls, and technical or procedural concerns specifically, the outcome was classified according to whether patients were able to initiate, resume or finalize their treatments without interruption of their prescribed schedule. Finally, we recorded the frequency, necessity and indication of a nurse’s home-visit, patients’ presentation at the practice or acute hospitalization.

### Analysis

The ‘reporting of studies conducted using observational routinely-collected health data’ (RECORD)-statement was acknowledged [18]. All statistical analyses were performed using SPSS Statistics version 26 (IBM Corp., Armonk, New York, USA) and the R environment for statistical computing [19].

## Results

### Overview of all calls documented during the studies’ observational period

**Fig. 1:**
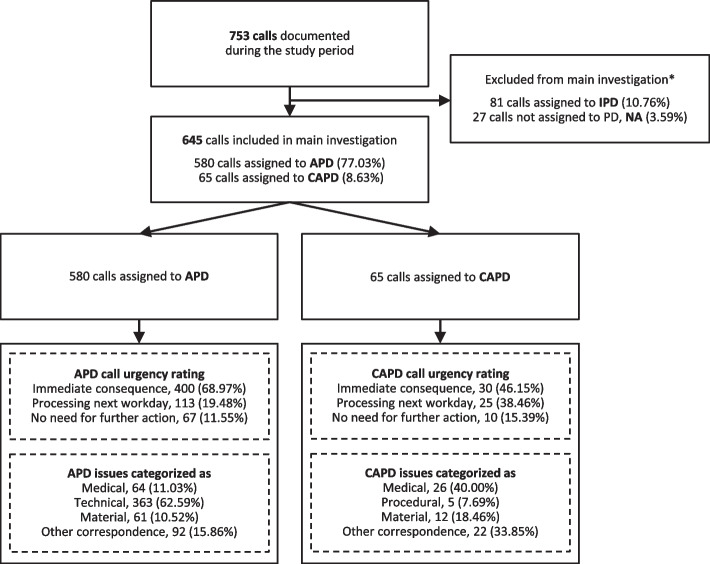
Flow chart of total calls, assignment to PD-modalities (automated peritoneal dialysis [APD], continuous ambulatory peritoneal dialysis [CAPD]), grading of call urgency and categorization to technical-, medical- or material-related issues or other correspondence

In the observational time frame of 2557 consecutive on-duty days, a total of 753 calls were documented, evaluated and assorted to 34 items reflecting the major call-issue in five categories. This accounted for about one call in every 29.45% of shifts. Of those 580 calls were associated with APD (77.03%), 65 with CAPD (8.63%), 81 with IPD (10.76%) while 27 were not assigned to a PD modality (3.59%) (Fig. 1). The latter comprised patients that were not yet or not anymore doing PD or others calling regarding unrelated issues. In total 123 persons were doing PD in the observational time frame at our centre. Of those, 85 (69.1%) called the PD support service at least once outside of standard office hours. The mean age of these patients at commencement of PD was 69 years (56–77, 25-75^th^ CI), 32 (37.65%) were female. Typical distribution of PD modalities in our centre was varying, approximating 66.86% (SD 8.19) doing APD, 28.37% (SD 9.61) doing CAPD and 4.77% (SD 1.8) being treated with IPD, yet there was a trend for more persons on APD over CAPD calling the support service. Time of call was documented for 674/753 calls (89.51%). The time-dependent analysis was split into ante meridiem (a.m.) and post meridiem (p.m.). The majority of calls were registered around 7:30 a.m. (5:00–9:00; median, 25-75^th^ CI) in the morning and around 6:30 p.m. (5:00–8:15) in the evening (Figure S1).

Calls not assigned to a category (NA) and those categorized to IPD were excluded from the main investigation since these calls coming from the clinic or hospital personnel were not related to home-dialysis associated issues. Further information on these calls is provided in the supplemental material.

### Assessment of call urgency among all calls assigned to APD and CAPD

In 66.7% of combined APD and CAPD calls the issues raised had an “immediate consequence” and were predominantly technical issues. In 21.4% of calls processing of the issue on the next working day was sufficient. The attributed call urgency according to PD-type and category is shown in Fig. 1 and Table S1. In 35 cases a home visit by the nurse or a patients’ visit at the practice was requested at the earliest possible time for further diagnostics. These mainly related to suspected peritonitis, suspected exit site infection or cycler software/handling issue not resolved by call (Table S2).

### Assessment of treatment continuity for APD and CAPD

We identified calls with issues that compromised successful continuation of prescribed treatment due to technical/procedural-, medical- or material-related issues. In 77.48% (320/413) patients were able to initiate, resume or finalize their treatment after phone consultation. Considering guidance or advice to manually resume the therapy with a last-bag exchange the rate increased to 83.54% (+ 6.05%). In 16.46% patients were not able to resume their treatment or had no last-bag option available (Table S3a).

Considering technical (APD) or procedural (CAPD) issues only, 75.0% (267/356 calls) resulted in successful initiation, resumption or finalizing of the prescribed treatment (Table S3b) and increased to 82.02% (+ 7.02%) using last-bag options. In 17.98% patients were not able to resume treatment due to technical errors, compliance issues or having no last-bag option available.

### Results assorted to call categories derived from patients’ chief complaint (APD and CAPD)

**Fig. 2:**
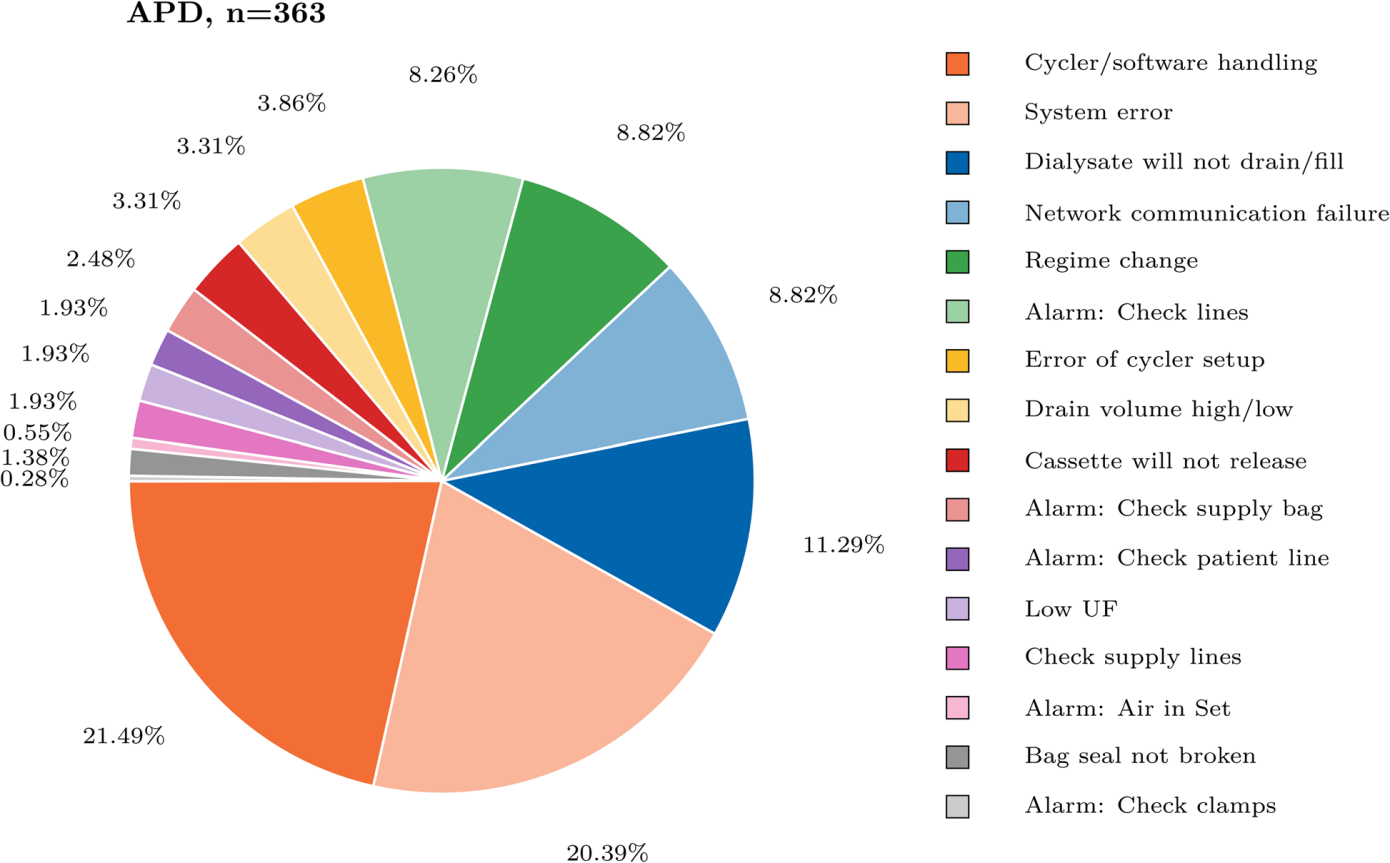
Proportional overview of all categorized technical issues in automated peritoneal dialysis home therapy (APD)

The majority of APD-associated calls (62,59%, 363/580) were related to category 1), technical issues. These were predominantly associated with cycler/software handling, system errors and patients reporting that dialysate would not drain or fill. An overview of all technical issues and errors for APD is provided in Fig. 2, Figure S2 and Figure S3.

**Fig. 3:**
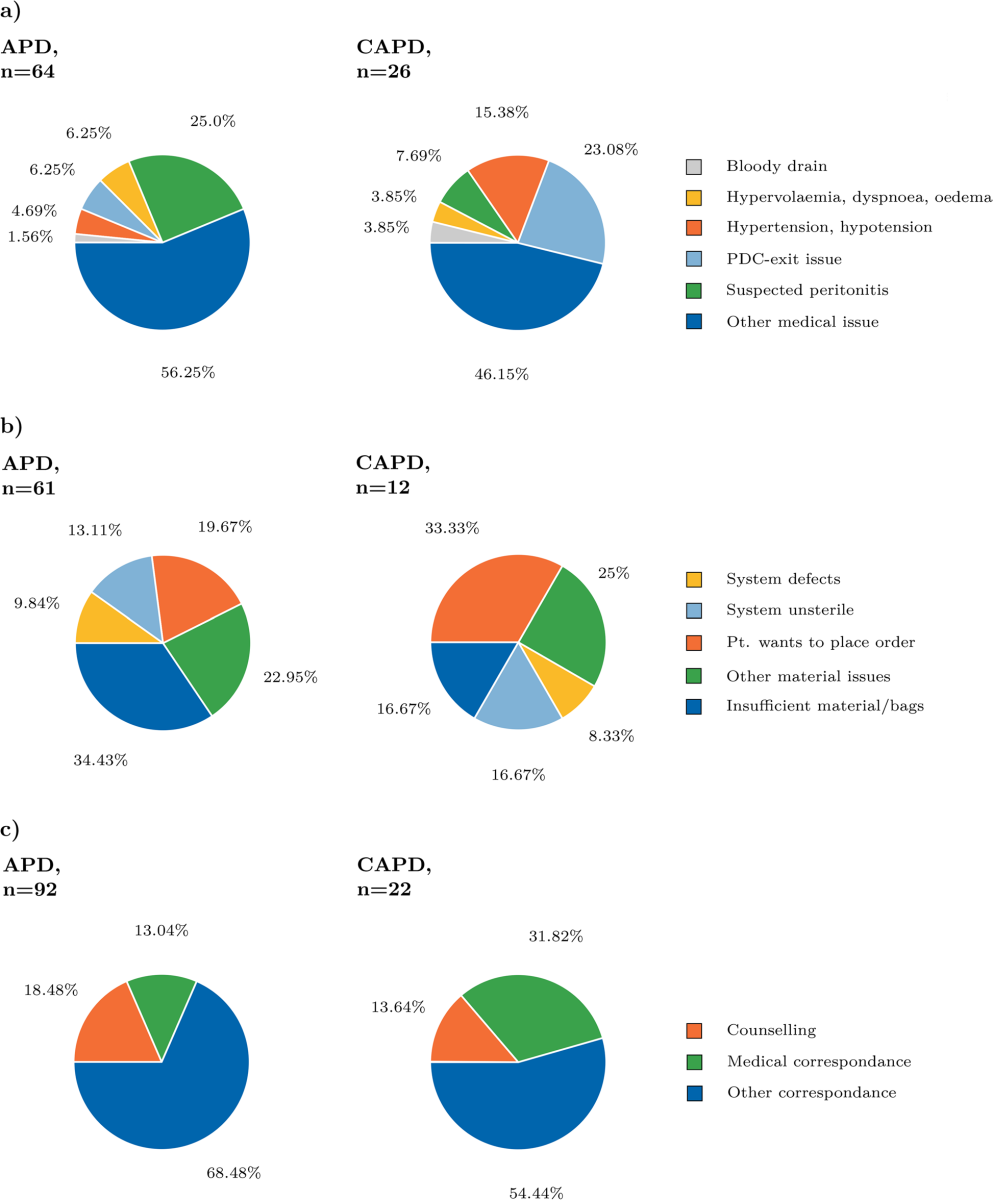
Proportional overview of calls categorized as **a** Medical-related, **b** Material-related issues as well as **c** Correspondence in automated peritoneal dialysis home therapy (APD) and continuous ambulatory peritoneal dialysis (CAPD). Abbreviations: PDC, PD catheter

In total, 90 calls of APD and CAPD (13.95%) were related to category 2, medical issues. The majority of these had an “immediate consequence” (61.1%, Table S1). In 20.0% of medical issues acute peritonitis was suspected (2.8% of total assessed calls). Others included symptoms associated with decompensated volume overload such as “hypervolemia, dyspnea, oedema” (5.6% of medical) and “hypertensive or hypotensive blood pressure” (7.8% of medical) or medical issues associated with the PD-catheter exit-site (11.1% of medical). An overview of all medical issues is provided in Fig. 3a.

Acute hospitalization of PD home-care patients was required in seven medical category-cases, only that were mainly related to suspected infections such as peritonitis (1.09% of total assessed calls, Table S4).

Material related issues (category 3, 73/645, 11.32%, Fig. 3b), exemplary included material leakage or material handling errors resulting in an unsterile PD catheter transfer set. The majority of calls, however, were associated with “insufficient material/bags” or patients wanting to order follow-up supplies in the out-of-office hours that involved “processing next working day” (Table S1).

Other correspondence (category 4) was filed as “medical correspondence” when patients were hospitalized, hospital staff or outpatient practices wanted to discuss current acute or non-acute PD-patients’ medical findings or medical proceedings (Fig. 3c). Only 14.9% of these calls had “immediate consequence” and 50.9% had “no need for further action” (Table S1). Patients also called for counselling reasons. Other calls were documented as non-acute correspondence, such as but not limited to confirmation of follow-up visits, patients needing medical prescription or attestations, but also patients checking if the service-number was still working properly, patients telling they dialled the wrong number or patients wanting to say good night to the team.

An overview of all calls documented during the observational period assigned to the respective categories is provided in Table S5.

## Discussion

In this retrospective study, we assessed and provide data on telephone calls to a nurse-provided on-call emergency PD support service in an outpatient PD reference centre comprising 753 categorized calls over a seven-year timeframe. Calls were made mainly in the early evening and morning hours. We found that two thirds of calls had immediate consequence to be handled by the PD nurse on-call duty. The vast majority was associated with technical- followed by medical and material issues. In up to 84% nurses were able to guide the patients to successfully resolve the issue and guide callers to initiate, resume or finalize their treatment. In a minority of calls (6.5%) the patient was asked to urgently present to the PD centre or hospital emergency ward for timely initiation of treatment such as in suspected peritonitis or PD-catheter associated infections, leakage, blood pressure deviations or volume overload.

There is a growing interest in promoting home-based renal replacement therapies [20]. Although 24 h on-call PD support is recommended [5, 6], no previous literature assessed the nature of emergencies challenging an on-call PD support service.

Reintjes et al*.* filed 172 calls in an on-call patient support for a home haemodialysis program during a 4-month period [16]. The majority of calls (> 57%) were also related to various aspects of technical issues or machine setup troubleshooting [16].

In the present study, calls were predominantly made by APD patients requiring technical support in the APD-setup, with subsequent errors in follow-up cycles and finalizing phases. These were caused by incorrect operation, uncertainties in handling the supplies or set, software or confirmation of a new treatment programs. The Claria sharesource remote connectivity provides detailed overview of the therapy performed and ability to change the treatment prescription, however, data only synchronizes to the cloud when the APD session is successfully finalized. Thus, there is no possibility to investigate errors in ongoing treatments. Modem network connectivity problems were predominantly registered with older 3G-modems that were later replaced with 4G networking, which reduced the frequency of connectivity failure.

While setting up a therapy session, specifically, prompting to “check lines” or venting the set prevented cyclers to commence therapy. However, after program initiation, the cycler prompts to “check lines” may ultimately correspond to the same underlying issue as “check supply lines” or “check supply bags” in the setup phase. During treatment, the cycler will no longer differentiate between the direct source of line-obstacle. Therefore, also insufficient venting caused by bags’ mis- or disconnection may be a cause of program interruption. This error, however, may also relate to insufficient amount of dialysate in case of frequent cycle interruptions. In such cases, we suggest withholding approximately 500 mL of the total dialysate for flushing of lines if required.

We suggest that more detailed specification of error messages is necessary to simplify machine use by the person doing PD. Recent Claria cycler software provides the option to bypass low initial drain volume errors which are frequently reported when patients start their therapy with empty cavity, but the program demands for a prespecified initial drain (exemplary in last-bag prescriptions).

Errors reported after midnight were usually associated to the filling phase 3 of 5 when the dialysate from the secondary bag is recruited. These are frequently due to bag connection problems. Similar problems may also occur when using a last-bag for a daily dwell.

At the end of treatment patients issued “low UF” (ultrafiltration) errors, which are displayed when the accumulated UF for the session is below a prespecified target-UF at the end of the last regular drain. Instructing the patient to sit or stand up may recruit more abdominal fluid and facilitate the drain.

Serious adverse events in home haemodialysis are usually acute, but uncommon [14]. Patients and providers, however, cite fears of catastrophic events and general lack of structures as a barrier for the uptake of PD as a home-based renal replacement therapy [10]. However, for example, preventive measures, education and early invited visits to the PD centre for inspection and treatment, if necessary, may prevent exit site infections from progression to tunnel infections or peritonitis [21]. Cloudy or bloody dialysate as well as abdominal pain is oftentimes registered at drain. We request our patients to take samples from the drain bag for diagnostic and present to the centre for a physical, laboratory examination and initiation of therapy when indicated. Rare, acute or rapidly worsening conditions are directed to go to the emergency department.

In cases of system errors discontinuing the conventional program cycle, patients in our centre are instructed to either switch off the cycler and disconnect in the morning or safely disconnect if they feel confident about it, rather than change bags or reconnect lines or bags to the cycler in ongoing treatments, as this may compromise setup integrity and asepsis [22]. Depending on the fill volume, we advise to use a drain bag and manual last-bag after getting up in the morning.

Aside from medical aspects, calls may be made for counselling or out of subjective insecurities. This psychosocial support, however, may reassure patients’ confidence and is of major relevance for patients’ acceptance of PD and thus a cornerstone of provided treatment quality and patients’ quality of life [23].

Our findings have several important implications. The absence of an on-call support to address clinical or technical issues would potentially result in missed or postponed treatments affecting quality and achievement of therapy goals. Providing care specialized and experienced in PD, especially considering technical issues and acute medical complications requires the provision of emergency structures throughout the year, 24 h a day, seven days a week [24, 25]. This enabled our patients in the vast majority of issues to continue therapy quickly and safely underscoring the need and ability of nurses to triage such cases. Additionally, communication skills and knowledge of the patient’s medical courses and their physical home environments may facilitate ability to respond to and resolve patients’ concerns and issues [26]. We suggest that timely patient-centred, and effective support may be substantial for patients’ clinical outcome and technique survival potentially preventing progression of adverse complications.

However, often lack of training, expertise and experience of nurses and doctors may prevent the establishment or advancement of home therapy [9, 10]. The development of a good training program for patients and PD nurses to perform on-call duties and providing technical support through other means is needed. Digital resources may be helpful [27], however the communication skills and practical experience of a PD nurse guiding a person via telephone is essential for the interventions’ success [26]. Therefore, not only do we need to implement interprofessional home dialysis qualification, education and practice -but also, complementary, novel multidisciplinary outpatient and inpatient structures in order to safely care for patients with home-based treatment [28–30].

This observational study was part of a quality improvement program, using routinely collected health data from medical records that documented on-call support service incidents in a representative outpatient PD-cohort. As a limitation, not all incidents may have been documented with similar detail. External validity and representativeness may therefore be limited. Categories and issue information were assigned to best match the staffs’ shift protocols. To address potential confounding, call protocols were reviewed and matched by three experienced readers in order to reflect the concerns of our patients to the best of judgement. Finally, this is a single centre retrospective study so the results should be considered as preliminary.

As there is continued interest to increase the number of patients treated with home dialysis [31], more patients will be tasked with performing complex procedures at home. A key aspect of providing the treatment support availability is the reduction of patients’ uncertainties [32]. The on-call PD nurses’ triage should aim primarily at minimizing risks to patients’ health. Notably, the majority of calls were related to technical issues associated with APD. Accordingly, improvement of usability and simplification of APD-systems is needed. Further tackling of error-proneness and the simplification of technology and software of APD-systems may improve handling in homecare programs, continuity of care, promote the acceptance and spread of PD, reduce the workload of employees and cut down on the number of calls [33]. Especially for the persons using dialysis machines daily, developments in home monitoring and telemedicine may facilitate such procedures and further improve quality of life and support options [32].

## Conclusion

Maintaining quality PD homecare, the provision of trained personnel is indispensable, and the information gathered in this paper may be used as a foundation to tailor educational programs for nephrology nurses and doctors to further develop their competencies in PD. This is underlined by the fact that more than two thirds of calls to our PD service had immediate consequences and needed guidance or intervention provided by nurses. Their intervention reduced the disruption to prescribed treatment cycles suggesting improvement of treatment quality. In a small proportion of calls, however, most relevant to patients’ safety, the on-call PD nurses responded to acute conditions and facilitated rapid diagnostics and therapy﻿.

### Supplementary Information

**Supplementary Material 1. Figures S1-S3, Tables S1-S5.**

## Data Availability

The datasets generated and/or analysed during the current study are not publicly available due to restrictions specified in the study protocol but are available from the corresponding author on reasonable request.

## Abbreviations

APD: Automated peritoneal dialysis
CAPD: Continuous ambulatory peritoneal dialysis
IPD: Intermittent peritoneal dialysis
PD: Peritoneal dialysis
