# Supplementary material for: Analysis of a nurse-provided on-call peritoneal dialysis support in an outpatient reference care centre
Source: BMC Nurs. 2024 Mar 1;23:144. doi: 10.1186/s12912-024-01812-4 (PMC10905825; doi:10.1186/s12912-024-01812-4)
Supplement: Supplementary file 1 — Supplementary Material 1. Figures S1-S3, Tables S1-S5. [file 12912_2024_1812_MOESM1_ESM.pdf]

Analysis of a nurse-provided on-call peritoneal dialysis support in an outpatient reference care center

Annemarie Albert[1,2], Stefan Richter[1], Philipp Kalk[1], Philipp Stieger[3], Rainer Peter Woitas[1], Rüdiger C. Braun-Dullaeus[3], Christian Albert[1,3,4]

[1] Diaverum Renal Services, Potsdam, Germany;

[2] Department of Nephrology and Endocrinology, Klinikum Ernst von Bergmann, Potsdam, Germany;

[3] University Clinic for Cardiology and Angiology, Medical Faculty, Otto-von-Guericke University, Magdeburg, Germany

[4] Department of Nephrology, Central Clinic Bad Berka, Bad Berka, Germany

Corresponding author:

Christian Albert, MD, University Clinic for Cardiology and Angiology, Medical Faculty, Otto-von-Guericke University, Magdeburg, Leipziger Str. 44, 39120 Magdeburg, Germany

E-Mail: `Christian.Albert@med.ovgu.de`

Supplemental material

Figure S1 a-b

Overview of frequency and time distribution of calls from 2015-2021, Time of call was recorded for N=674 calls (89.5%). Time was not filed for N=79 calls (10.5%).

Figure S2

Error codes filed in automated peritoneal dialysis home therapy (APD) and intermittent automated peritoneal dialysis (IPD) on the clinic ward or intensive care unit. Predominantly system error 1032 (associated with overvoltage protection) and system error 2240 (air in set) were queried.

Figure S3

Proportional overview of all categorized technical issues in automated peritoneal dialysis home therapy (APD) and intermittent peritoneal dialysis (IPD) performed on the clinic ward or intensive care unit.

Table S1 a-b

Distribution of call urgency ratings (Immediate consequence, processable next working day or no need for further action) for calls performed by or referring to a) automated peritoneal dialysis (APD), continuous ambulatory peritoneal dialysis (CAPD), intermittent automated peritoneal dialysis (IPD) and unassigned patients; b) referring to respective category. For this evaluation, category (5) calls are shown separately as they were associated with timely planning and performing assisted IPD or perioperative care in the teaching hospital by the PD team and therefore these calls were considered to have an immediate consequence.

Table S2

Overview of patients' indication for nurse home visit or patients' practice visit

Table S3 a-b

Overview of calls with issues that potentially compromised successful continuation of prescribed treatment due to (a) technical, procedural, medical or material-related issues and (b) technical or procedural issues, only - for automated peritoneal dialysis (APD), continuous ambulatory peritoneal dialysis (CAPD) and intermittent automated peritoneal dialysis (IPD).

Table S4

Overview of patients' indication for acute hospitalization

Table S5

Summary of all calls filed from 2015-2021 assorted to 34 subsections of technical-, medical- and material-related issues or other correspondence performed by or referring to automated peritoneal dialysis (APD), continuous ambulatory peritoneal dialysis (CAPD), intermittent automated peritoneal dialysis (IPD) and unassigned callers or patients. These comprise patients that were not yet or not anymore doing PD or other callers/personel that did not call in favor of PD patients.

Figure S1 a. Call distribution ante meridiem (a.m.), N=222

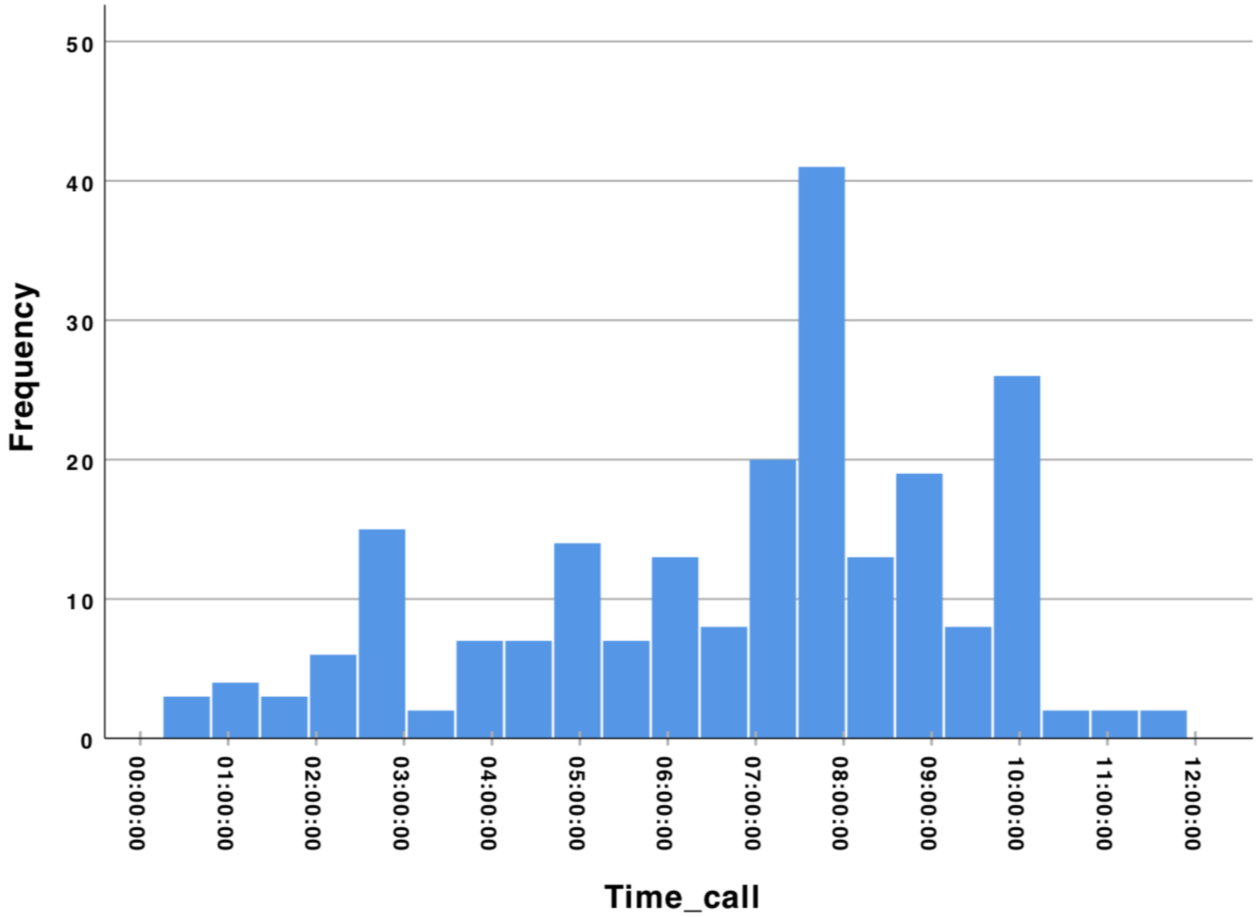

Figure S1 b. Call distribution post meridiem (p.m.), N=452

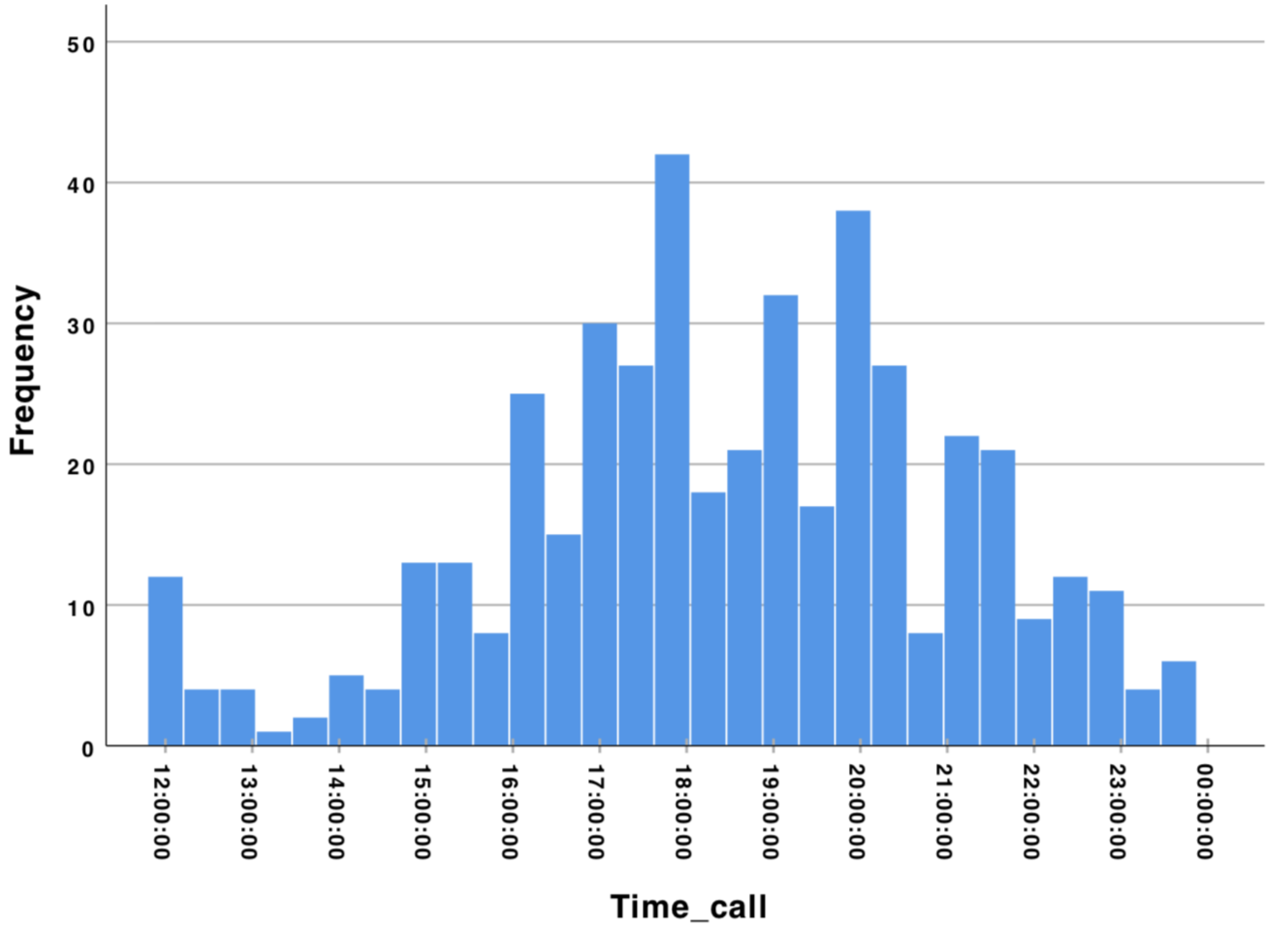

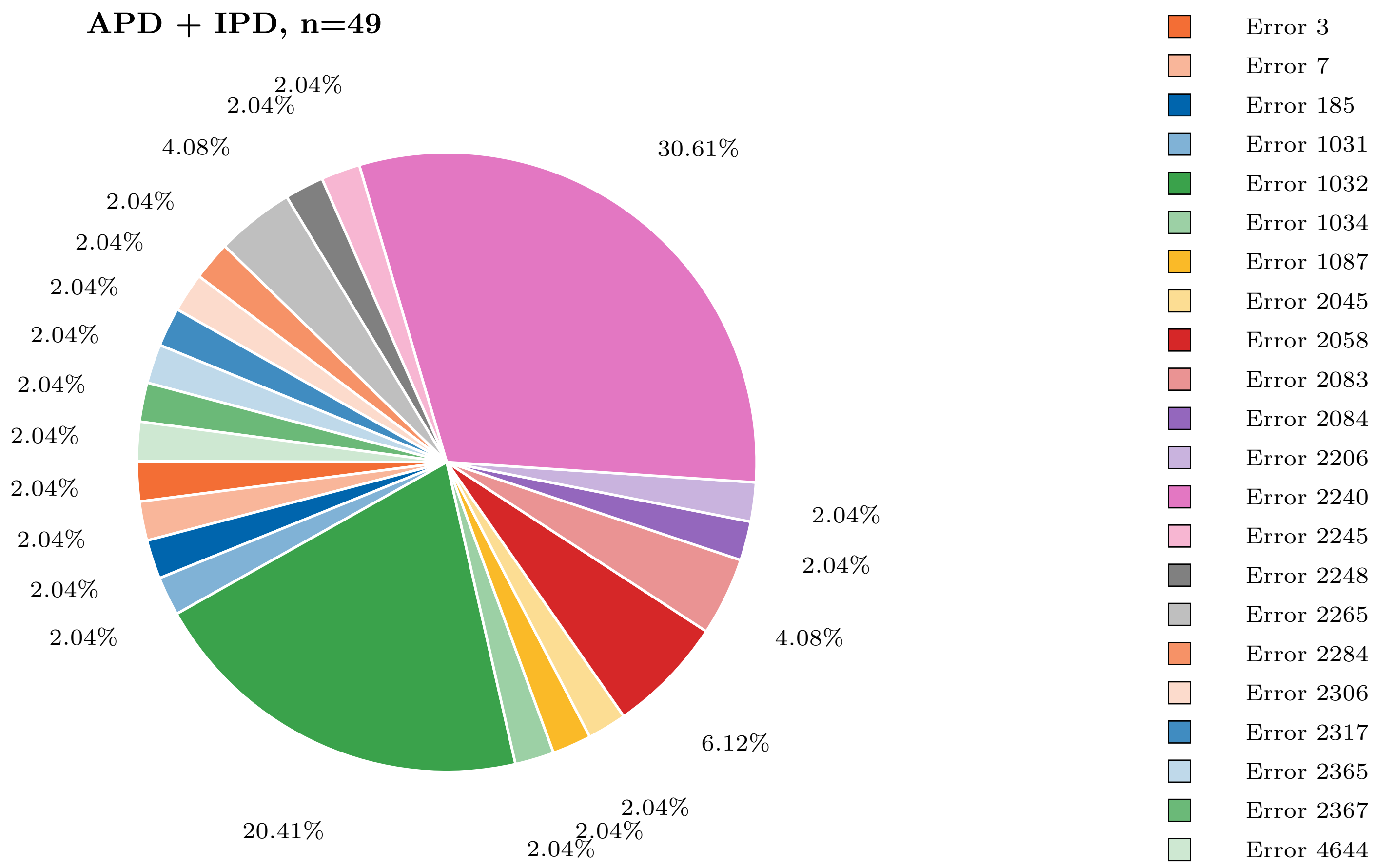

Figure S2

Errors filed in automated peritoneal dialysis home therapy (APD) and intermittend automated peritoneal dialysis (IPD) on the clinic ward or intensive care unit. Predominantly ■ system error 1032 (associated with overvoltage protection) and ■ system error 2240 (air in set) were queried by the patients.

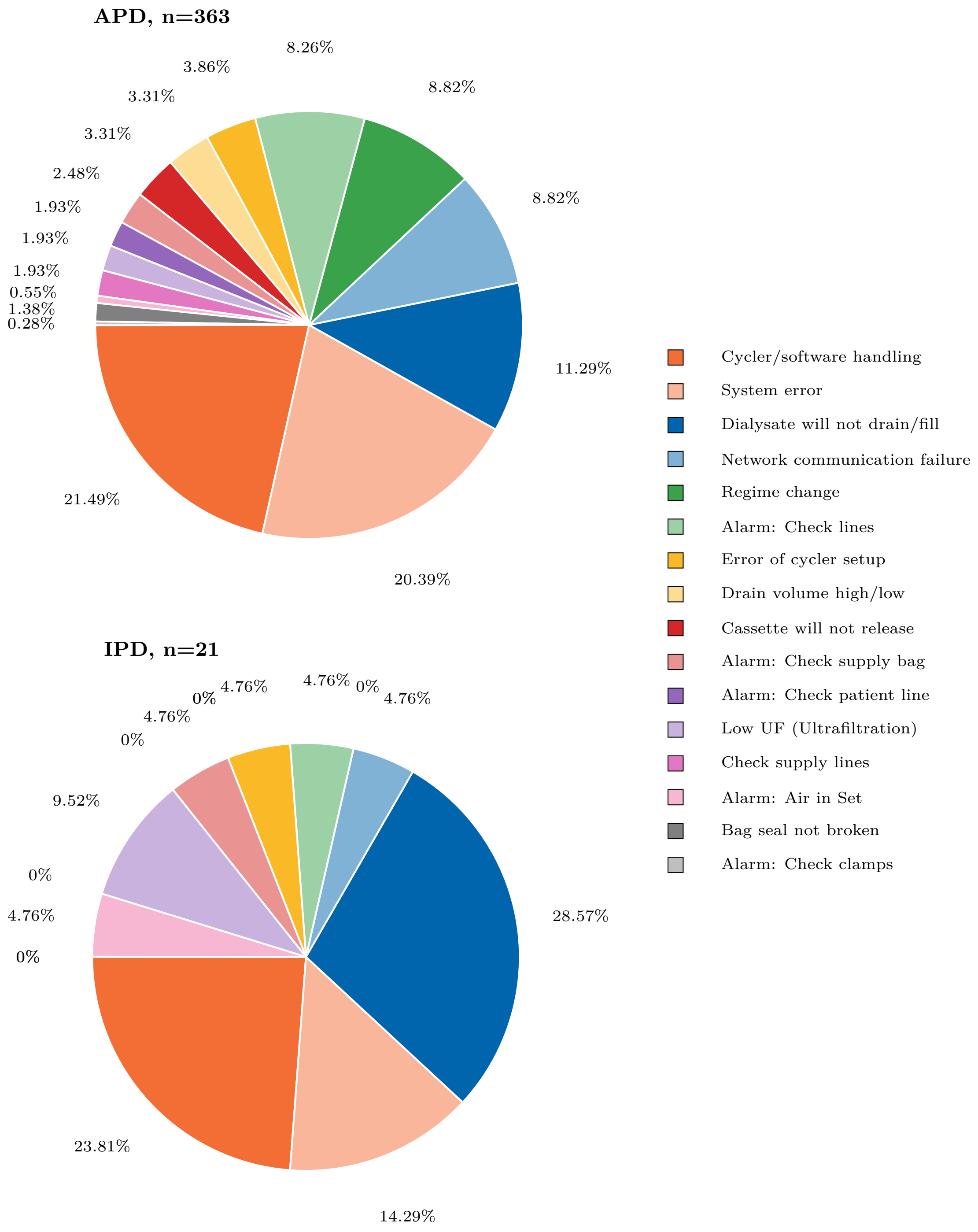

Figure S3

Proportional overview of all categorized technical issues in automated peritoneal dialysis home therapy (APD) and intermittent peritoneal dialysis (IPD) performed on the clinic ward or intensive care unit.

Table S1a. Call urgency rating, summary and percentage according to PD-type

Category	Total calls, N (%)	APD, N (% of APD, % of total)	CAPD, N (% of CAPD, % of total)	IPD, N (% of IPD, % of total)	unassigned, N (% of unassigned, % of total)
Immediate consequence	504 (66.93)	400 (68.97, 53.12)	30 (46.15, 3.98)	71 (87.65, 9.43)	3 (11.11, 0.40)
Processing next working day	157 (20.85)	113 (19.48, 15.01)	25 (38.46, 3.32)	4 (4.94, 0.53)	15 (55.56, 1.99)
No need for further action	92 (12.22)	67 (11.55, 8.90)	10 (15.38, 1.33)	6 (7.41, 0.80)	9 (33.33, 1.20)
Sum	753 (100)	580.00 (100, 77.03)	65 (100, 8.63)	81.00 (100, 10.76)	27.00 (100, 3.59)

Table S1b. Call urgency rating, summary and percentage according to call category type

Category	Total calls, N (%)	technical, N (% of technical, % of total)	medical, N (% of medical, % of total)	material, N, (% of material, % of total)	correspondance, N (% of correspondance, % of total)	PD in hospital (% of total)
Immediate consequence	504 (66.93)	350 (89.97, 46.48)	57 (61.29, 7.57)	28, (35.00, 3.72)	23 (15.97, 3.05)	47 (6.24)
Processing next working day	157 (20.85)	30 (7.71, 3.98)	32 (34.41, 4.25)	44, (55.00, 5.84)	50 (34.72, 6.64)	0 (0.00)
No need for further action	92 (12.22)	9 (2.31, 1.20)	4 (4.30, 0.53)	8, (10.00, 1.06)	71 (49.31, 9.43)	0 (0.00)
Sum	753 (100)	389 (100, 51.66)	93 (100, 12.35)	80, (100, 10.62)	144 (100, 19.12)	47 (6.24)

Table S2. Overview of patients' indication for nurse home visit or patients' practice visit

Year	Type of PD	Category	Reason	Grouped as
2021	CAPD	Medical	Suspected worsening of exit site infection	PD catheter exit site issue
2021	CAPD	Medical	Suspected exit site infection	PD catheter exit site issue
2021	APD	Technical	System error; Cylcler exchange at the practice	System error
2019	APD	Technical	System error; Cylcler exchange at the practice	System error
2019	APD	Technical	Cycler software/handling issue not resolved by call	Cycler handling
2019	APD	Technical	System error; Cylcler exchange at the practice	System error
2018	APD	Technical	Cycler software/handling issue not resolved by call	Cycler handling
2018	APD	Material	Insufficient material to perform PD	Insufficient material
2017	APD	Medical	Suspected exit site infection	PD catheter exit site issue
2017	APD	Technical	Cycler handling issue not resolved by call	Cycler handling
2017	APD	Technical	Cycler handling issue not resolved by call	Cycler handling
2017	APD	Technical	Cycler handling issue not resolved by call	Cycler handling
2017	APD	Technical	Cycler handling issue not resolved by call	Cycler handling
2017	APD	Medical	Exit site issue after recent PDC implantation	PD catheter exit site issue
2017	APD	Medical	Suspected peritonitis	Suspected peritonitis
2017	CAPD	Medical	Suspected peritonitis	Suspected peritonitis
2017	CAPD	Medical	Suspected peritonitis	Suspected peritonitis
2017	CAPD	Medical	Suspected peritonitis	Suspected peritonitis
2017	APD	Medical	Exit site issue after PDC implantation	PD catheter exit site issue
2016	APD	Technical	Cycler software/handling issue not resolved by call	Cycler handling
2016	APD	Technical	Cycler software/handling issue not resolved by call	Cycler handling
2016	APD	Technical	Cycler software/handling issue not resolved by call	Cycler handling
2016	APD	Technical	Cycler software/handling issue not resolved by call	Cycler handling
2016	APD	Technical	Cycler software/handling issue not resolved by call	Cycler handling
2016	APD	Technical	Cycler software/handling issue not resolved by call	Cycler handling
2016	APD	Technical	Cycler software/handling issue not resolved by call	Cycler handling
2016	APD	Material	Insufficient material to perform PD	Insufficient material
2016	APD	Technical	Dialysate would not drain, not resolved by call	Dialysate would not drain
2016	CAPD	Medical	Suspected peritonitis	Suspected peritonitis
2016	CAPD	Medical	Suspected peritonitis	Suspected peritonitis
2015	CAPD	Medical	Suspected peritonitis	Suspected peritonitis
2015	CAPD	Material	Unsterile catheter connection	Unsterile system
2015	CAPD	Medical	Exit site, dialysate leakage	PD catheter exit site issue
2015	APD	Medical	Suspected peritonitis	Suspected peritonitis
2015	APD	Medical	Suspected peritonitis	Suspected peritonitis

APD, automated peritoneal dialysis; PD, peritoneal dialysis, PDC, PD catheter

Table S3a. Continuation or successful finalization of therapy over all sections (technical, procedural, medical, material)

Category	Total calls, N (%)	APD, N (% of APD, % of total)	CAPD, N (% of CAPD, % of total)	IPD, N (% of IPD, % of total)
Continue	384 (79.83)	301 (76.40, 62.58)	19 (100.00, 3.95)	64 (94.12, 13.31)
Lastbag	25 (5.20)	25 (6.35, 5.20)	NA (NA, NA)	0 (0.00, 0.00)
Discontinue	72 (14.97)	68 (17.26, 14.14)	0 (0.00, 0.00)	4 (5.88, 0.83)
Continue or Lastbag	409 (85.03)	326 (82.74, 67.78)	19 (100.00, 3.95)	64 (94.12, 13.31)
Sum	481 (100.00)	394 (100.00, 81.91)	19 (100.00, 3.95)	68 (100.00, 14.14)

Table S3b. Continuation or successful finalization of therapy when reporting a technical or procedural issue for APD, CAPD or IPD patients

Category	Total calls, N (%)	APD, N (% of APD, % of total)	CAPD, N (% of CAPD, % of total)	IPD, N (% of IPD, % of total)
Continue	284 (75.33)	262 (74.64, 69.50)	5 (100.00, 1.33)	17 (80.95, 4.51)
Lastbag	25 (6.63)	25 (7.12, 6.63)	NA (NA, NA)	0 (0.00, 0.00)
Discontinue	68 (18.04)	64 (18.23, 16.98)	0 (0.00, 0.00)	4 (19.05, 1.06)
Continue or Lastbag	309 (81.96)	287 (81.77, 76.13)	5 (100.00, 1.33)	17 (80.95, 4.51)
Sum	377 (100.00)	351 (100.00, 93.10)	5 (100.00, 1.33)	21 (100.00, 5.57)

Table S4. Overview of patients' indication for acute hospitalization

Year	Type of PD	Category	Reason	Grouped as
2021	APD	Medical	Circulatory disturbance	Hypotension/hypertension/hypervolaemia
2020	APD	Medical	Suspected peritonitis	Suspected peritonitis
2018	APD	Medical	Suspected myocardial infarction	Hypotension/hypertension/hypervolaemia
2018	APD	Medical	Suspected peritonitis	Suspected peritonitis
2017	NA	Medical	Transplant alert	Other medical issue
2017	APD	Medical	Hypervolaemia, uncontrolled weight gain	Hypotension/hypertension/hypervolaemia
2016	APD	Medical	Suspected sepsis, diabetic foot	Other medical issue
2016	APD	Medical	Convulsion/seizure	Other medical issue

APD, automated peritoneal dialysis; PD, peritoneal dialysis; NA, not assigned (call referred to in-centre haemodialysis patient)

Table S5: Summary and percentage of total calls documented during observational period according to PD-modality; N=753

Categories	Total calls, N	of total, %	APD. N	% of APD	% of total	CAPD. N	% of CAPD	% of total	IPD. N	% of IPD	% of total	unassigned. N	% of unassigned	% of total
**Technical** (APD,IPD)**/ Procedural** (CAPD)														
Dialysate will not drain or fill	47	6.24	41	7.07	5.44	0	0.00	0.00	6	7.41	0.80	0	0.00	0.00
Cassette will not release	12	1.59	12	2.07	1.59	0	0.00	0.00	0	0.00	0.00	0	0.00	0.00
Alarm: Check lines	31	4.12	30	5.17	3.98	0	0.00	0.00	1	1.23	0.13	0	0.00	0.00
Alarm: Check supply lines	7	0.93	7	1.21	0.93	0	0.00	0.00	0	0.00	0.00	0	0.00	0.00
Alarm: Check supply bag	10	1.33	9	1.55	1.20	0	0.00	0.00	1	1.23	0.13	0	0.00	0.00
Alarm: Air in set	6	0.80	5	0.86	0.66	0	0.00	0.00	1	1.23	0.13	0	0.00	0.00
Drain volume high/low	12	1.59	12	2.07	1.59	0	0.00	0.00	0	0.00	0.00	0	0.00	0.00
Low ultrafiltration (Uf)	9	1.20	7	1.21	0.93	0	0.00	0.00	2	2.47	0.27	0	0.00	0.00
Alarm: Check patient line	7	0.93	7	1.21	0.93	0	0.00	0.00	0	0.00	0.00	0	0.00	0.00
Alarm: Check clamps	1	0.13	1	0.17	0.13	0	0.00	0.00	0	0.00	0.00	0	0.00	0.00
Cycler/software handling	83	11.02	78	13.45	10.36	0	0.00	0.00	5	6.17	0.66	0	0.00	0.00
Regime change	32	4.25	32	5.52	4.25	0	0.00	0.00	0	0.00	0.00	0	0.00	0.00
Errors of cycler setup	15	1.99	14	2.41	1.86	0	0.00	0.00	1	1.23	0.13	0	0.00	0.00
Bag seal not broken	2	0.27	2	0.34	0.27	0	0.00	0.00	0	0.00	0.00	0	0.00	0.00
Network communication failure	33	4.38	32	5.52	4.25	0	0.00	0.00	1	1.23	0.13	0	0.00	0.00
System error	77	10.23	74	12.76	9.83	0	0.00	0.00	3	3.70	0.40	0	0.00	0.00
CAPD procedural assistance	5	0.66	0	0.00	0.00	5	7.69	0.66	0	0.00	0.00	0	0.00	0.00
**Medical**														
Suspected peritonitis	19	2.52	16	2.76	2.12	2	3.08	0.27	1	1.23	0.13	0	0.00	0.00
Boody drain	2	0.27	1	0.17	0.13	1	1.54	0.13	0	0.00	0.00	0	0.00	0.00
Hypervolaemia. dyspnoea. oedema	5	0.66	4	0.69	0.53	1	1.54	0.13	0	0.00	0.00	0	0.00	0.00
PDC-exit issue	10	1.33	4	0.69	0.53	6	9.23	0.80	0	0.00	0.00	0	0.00	0.00
Hypertension. hypotension	7	0.93	3	0.52	0.40	4	6.15	0.53	0	0.00	0.00	0	0.00	0.00
Other medical issue	50	6.64	36	6.21	4.78	12	18.46	1.59	1	1.23	0.13	1	3.70	0.13
**Material**														
Pt. wants to place order	16	2.12	12	2.07	1.59	4	6.15	0.53	0	0.00	0.00	0	0.00	0.00
System defects	7	0.93	6	1.03	0.80	1	1.54	0.13	0	0.00	0.00	0	0.00	0.00
Insufficient material/bags	27	3.59	21	3.62	2.79	2	3.08	0.27	3	3.70	0.40	1	3.70	0.13
Other material issues	20	2.66	14	2.41	1.86	3	4.62	0.40	0	0.00	0.00	3	11.11	0.40
System unsterile	10	1.33	8	1.38	1.06	2	3.08	0.27	0	0.00	0.00	0	0.00	0.00
**Correspondance**														
Medical correspondance	30	3.98	12	2.07	1.59	7	10.77	0.93	4	4.94	0.53	7	25.93	0.93
Other correspondance	94	12.48	63	10.86	8.37	12	18.46	1.59	4	4.94	0.53	15	55.56	1.99
Counselling	20	2.66	17	2.93	2.26	3	4.62	0.40	0	0.00	0.00	0	0.00	0.00
**PD in hospital**														
IPD performed (ward or ICU)	39	5.18	0	0.00	0.00	0	0.00	0.00	39	48.15	5.18	0	0.00	0.00
IPD flush (postoperatively)	3	0.40	0	0.00	0.00	0	0.00	0.00	3	3.70	0.40	0	0.00	0.00
Antibiotic bag infused	5	0.66	0	0.00	0.00	0	0.00	0.00	5	6.17	0.66	0	0.00	0.00
**Sum**	**753**	100.00	**580**	100.00	77.03	**65**	100.00	8.63	**81**	100.00	10.76	**27**	100.00	3.59
